# Diversity of endophytic fungi isolated from different plant parts of *Acacia mangium*, and antagonistic activity against *Ceratocystis fimbriata*, a causal agent of Ceratocystis wilt disease of *A. mangium* in Malaysia

**DOI:** 10.3389/fmicb.2022.887880

**Published:** 2022-11-08

**Authors:** Mohd Farid Ahmad, Rozihawati Zahari, Mastura Mohtar, Wan Azhar Wan-Muhammad-Azrul, Muhammad Syahmi Hishamuddin, Nik Iskandar Putra Samsudin, Affendy Hassan, Razak Terhem

**Affiliations:** ^1^Mycology and Pathology Unit, Forest Research Institute Malaysia, Kepong, Malaysia; ^2^Laboratory of Forest Pathology and Tree Health, Department of Forestry Science and Biodiversity, Faculty of Forestry and Environment, Universiti Putra Malaysia (UPM), Serdang, Malaysia; ^3^Bio Activity Programme, Natural Products Division, Forest Research Institute Malaysia, Kepong, Malaysia; ^4^Pest and Disease Management Programme, Horticulture Research Centre, Malaysian Agriculture Research and Development (MARDI), Persiaran Mardi-UPM, Serdang, Malaysia; ^5^Department of Food Science, Faculty of Food Science and Technology, Universiti Putra Malaysia (UPM), Serdang, Malaysia; ^6^Laboratory of Food Safety and Food Integrity, Institute of Tropical Agriculture and Food Security, Universiti Putra Malaysia (UPM), Serdang, Selangor, Malaysia; ^7^Faculty of Tropical Forestry, Universiti Malaysia Sabah, Kota Kinabalu, Sabah, Malaysia

**Keywords:** *Acacia mangium*, endophytic fungi, *Ceratocystis fimbriata*, Ceratocystis wilt, antagonism

## Abstract

*Acacia mangium* is an important wood for commercial products especially pulp and medium-density fibreboard. However, it is susceptible to *Ceratocystis fimbriata* infection, leading to Ceratocystis wilt. Therefore, the present work aimed to (i) establish the diversity of endophytic fungi in different plant parts of *A. mangium*,and (ii) evaluate the antifungal potentials of the isolated and identified endophytic fungi against *C. fimbriata*. Endophytic fungal identification was conducted by PCR amplification and sequencing of the internal transcribed spacer 1 (ITS1) and ITS4 regions of nuclear ribosomal DNA. A total of 66 endophytic fungi were successfully isolated from different parts of *A. mangium*; leaf (21), stem (13), petiole (12), root (9), flower (6), and fruit (5). The endophytic fungal isolates belonged to Ascomycota (95.5%) and Zygomycota (4.5%). For Ascomycota 13 genera were identified: *Trichoderma* (28.6%), *Nigrospora* (28.6%), *Pestalotiopsis* (12.7%), *Lasiodiplodia* (9.5%), *Aspergillus* (6.3%), *Sordariomycetes* (3%), and *Neopestalotiopsis*, *Pseudopestalotiopsis*, *Eutiarosporella*, *Curvularia*, *Fusarium*, *Penicillium*, and *Hypoxylon* each with a single isolate. For Zygomycota, only *Blakeslea* sp. (5%) was isolated. Against *C. fimbriata*, *Trichoderma koningiopsis* (AC 1S) from stem, *Nigrospora oryzae* (AC 7L) from leaf, *Nigrospora sphaerica* (AC 3F) from the flower, *Lasiodiplodia* sp. (AC 2 U) from fruit, *Nigrospora sphaerica* (AC 4P) from petiole, and *Trichoderma* sp. (AC 9R) from root exhibited strong inhibition for *C. fimbriata* between 58.33 to 69.23%. Thus, it can be concluded that certain endophytic fungi of *A. mangium* have the potential to be harnessed as anti-Ceratocystis agent in future biotechnological applications.

## Introduction

*Acacia mangium* Willd., a fast-growing and flowering leguminous tree native to Indonesia, Papua New Guinea, and Australia, has been introduced and cultivated into humid tropical lowland regions of Asia, South America, and Africa (Pinyopusarerk et al., 1993). In 1966, forest plantation of *A. mangium* began in Sabah, Malaysia, pioneered by D.I. Nicholson, an Australian forester. Commercial cultivation of *A. mangium* began in 1976 (Udarbe and Hepburn, 1986; Pinyopusarerk et al., 1993). The species was considered promising due to its stellar performance, superior growth, and multiple uses especially for pulp and medium-density fibreboard (Potter et al., 2006). Furthermore, pharmacological studies have also shown that the leaves of *A. mangium* exhibit antibacterial (Sarah Shafiei et al., 2017), antifungal (Mihara et al., 2005), antifilarial, and antihelmintic (Chaki et al., 2015) properties.

Despite its various commercial applications, *A. mangium* is susceptible to the infection of the ascomycetous pathogen, *Ceratocystis fimbriata*, which infects the wounds of *A. mangium* trees in plantations, and causes the Ceratocystis wilt disease (Kile, 1993; Roux and Wingfield, 2009; Tarigan et al., 2011; Brawner et al., 2015). Wounded tree caused by humans, other mammals including monkeys, elephants, squirrels or boring insects, and others factor such as wind, are likely to increase the disease spreading and tree mortality as the wound become the entrance for this Ceratocystis species to invade (Nasution et al., 2019). In Malaysia at the year of 2011, a severe case which was the first report of this disease infected approximately 40% of *A. mangium* trees in plantation at Tawau, Sabah. Later, this disease spreads to other regions on *A. mangium* plantation in Sabah such as Pitas, Kota Belud and Sipitang, where the incidence of this disease were about in range of 6–60% (Mandy and Wickneswari, 2014; Farid et al., 2018). Johor, Pahang and Sarawak were also reported faced the same disease problems to the *A. mangium* plantation in respective state. 50% out of 1,500 trees that were accessed in a 2-year-old *Acacia mangium* plantation in Johor have been infected by this disease (Farid et al., 2018). The main reason of this disease spreading and uncontrollable was due to lack of knowledge, researches and studies on how to overcome or prevent this disease to happen towards *Acacia mangium* trees (Lee, 2018).

There are no specific methods or guidelines established on how to handle this disease in Malaysia yet up to now. But there were several actions that commonly are used by the plantation managers to prevent the infection of this disease. As Ceratocystis species penetrate and invade the trees by wounds, this problems can be prevent by avoid the occurance of wound itself (Kile, 1993; Harrington, 2013; Nasution et al., 2019). Silviculture practice should be done in correct way and cautions. The timing of doing work for silviculture is also important to reduce the risk of disease development (Pilotti et al., 2016; Farid et al., 2018). Problems involved with wildlife in plantation areas also are count on in management such as establishment of wildlife management plan to overcome the conflicts occurred (Farid et al., 2018). Chemical control is one of application they used to delay the symptoms of the disease development and help the infected trees to live longer for at least 2 years (Blaedow, 2009; Nasution et al., 2019). Although the use of chemical fungicides are more preferred due to their rapid action, they are often associated with high production and application costs, human health hazards, restriction by domestic and international regulatory limits, trade bans, residual effects, environmental pollution, resistance development in pests, and potential elimination of beneficial natural enemies of the targeted pests (Yazid et al., 2020). Therefore, biological control is seen as a safer and cheaper alternative. Biological control is the use of living organisms (including microorganisms) to eliminate or reduce the density of pests / pathogens to safe levels (Wyckhuys et al., 2013). Often, indigenous organisms or microorganisms are utilised as biological control agent to minimise the risk of introducing foreign species that might grow uncontrollably and in turn become invasive. One such example of indigenous organisms or microorganisms is endophyte. The research is about using a microorganism (endophyte) to fight the pathogen (*Ceratocystis fimbriata*) which is one of biological control.

Like many other plant species, *A. mangium* is also associated with endophytes. Endophytes are usually bacteria or fungi that endosynbiotically live within a plant host without causing disease. These endophytes function to enhance the plant host growth and nutrient acquisition improve the plant host’s ability to tolerate abiotic stresses or decrease biotic stresses by enhancing the plant host’s resistance to infections (Farahat, 2020). Recently, an endophytic actinomycete of the genus *Fodinicola* was isolated from the roots of *A. mangium*, and has shown potential activity as a beneficial plant-growth promoter and specialised secondary metabolite producer (Phạm et al., 2020).

Despite endophytic fungi being regarded as new sources of novel bioactive compounds (Daouk et al., 1995; Cui et al., 2015), biological activities, and biotechnological developments, their true potential in controlling *A. mangium* diseases caused by *C. fimbriata* remains underexplored and underreported. Moreover, the leaf and root parts of *A. mangium* have been found to provide the habitats for various endophytic fungi (Mihara et al., 2005; Sarah Shafiei et al., 2017; Phạm et al., 2020). Nevertheless, besides leaf and root, other plant parts of the species should also be explored for endophytic fungi which might offer novel species or strains that possess valuable bioactive compounds useful in controlling the Ceratocystis wilt disease. Therefore, the objectives of the present work were (i) to establish the diversity of endophytic fungi in different plant parts of *A. mangium*, and (ii) to evaluate the antifungal potentials of the isolated and identified endophytic fungi against *C. fimbriata*.

## Materials and methods

### Plant materials

Ten seedlings of *Acacia mangium* (≈30–50 cm in height) and 2 *A. mangium* trees (≈30 cm in diameter at breast height) free from disease and insect infestation were randomly sampled, and identified at Serdang, Selangor (coordinate E 101^○^ 42.6333 N 2^○^ 59.1833). The root, stem, petiole, and leaf from healthy *A. mangium* seedlings were sampled in three replicates, respectively. In addition, three replicates of flower and fruit were also sampled from mature trees, respectively. Each plant part was cut into five 0.5 cm^2^ segments using a blade. These plant parts were washed thoroughly under running tap water to remove adherent debris on the surface.

### Isolation of endophytic fungi

Plant part segments were surface-sterilised following the protocol suggested by Nuangmek et al. (2021). Briefly, the plant part segments were washed thoroughly under running tap water, immersed in 70% ethanol (Cerilliant Corporation, United States) for 1 min, soaked in 4% NaOCl (Malay-Sino Chemical, Malaysia) for 1 min, rinsed thrice in sterile distilled water, and blot-dried using a sterile filter paper. Next, the surface-sterilised plant part segments were excised 1–2 mm from the edge, and explant-plated onto a Potato Dextrose Agar (PDA; Merck Milipore, Germany). The PDA plates were incubated at 27°C for 7 d. Single hyphae growing out from the cultivated plant part segments were sub-cultured onto fresh PDA. Pure cultures were grouped according to the six types of plant parts (root, stem, petiole, leaf, flower, and fruit). Isolates were group based on colour and morphology on PDA (Yoo and Eom, 2012). Cultures were maintained on PDA for 5 d before sub-cultured into Potato Dextrose Broth (PDB; Neogen®, United States) while shaken at 150 rpm at 26°C for 3–6 d. Following incubation, the culture supernatant was filtered through Whatman filter paper (Cytiva™ Sigma-Aldrich Chemie GmbH, Germany) before being used for genomic DNA extraction.

### DNA extraction and PCR amplification

A total of 100 mg of fungal mycelia harvested from PDB was used for fungal genomic DNA extraction. Fungal genomic DNA was extracted as previously described by Landum et al. (2016), in accordance with the manufacturer’s instructions, using the FAVORGEN Fungi/ Yeast Genomic DNA Extraction Mini Kit (Taiwan). The nuclear ribosomal DNA internal transcribed spacer (ITS) of the fungal isolates were amplified using the forward primer, ITS-F (5’-CTT GGT CAT TTA GAG GAA GTA A-3′) and the reverse primer, ITS4 (5’-TCC TCC GCT TAT TGA TAT GC-3′; White et al., 1990). The final reaction volume was 25 μl, containing 12.5 μl of 2X PCRBio Tag Mix Red (PCR Biosystems, UK), 0.4 μM of forward and reverse primers, and 10 mg of genomic DNA template. For negative control, the DNA was replaced with distilled water to verify the absence of contamination. The PCR was carried out using MyCycler™ (Bio-Rad, USA), programmed for 5 min at 95°C; 30 cycles for 30 s at 95°C, 30 s at 54.8°C, and 1 min at 72°C; and a final 10 min extension at 72°C. The PCR products were separated using 1% agarose gel in 1X TAE buffer (90 mM Tris-acetate and 2 nM EDTA, pH 8.0), stained with ethidium bromide (0.5 μg/ml), and visualised using FluorChem TM (Alpha Innotech, USA). The PCR products were sequenced by Apical Scientific Sdn. Bhd. (Malaysia). The sequences were deposited in NCBI GenBank, and compared with those already deposited in there *via* BLAST searches.

### Sequence and phylogenetic analyses

The resulting DNA sequences were aligned using MUSCLE software embedded in MEGA software version 10.0.5 (Kumar et al., 2018), and manually trimmed and edited to obtain the complete sequences. Homology searches were carried out using the BLAST program against the NCBI GenBank database.^1^ The Maximum Likelihood tree was constructed using MEGA software version 10.0.5 with all positions containing gaps and missing data were included for analysis. Clade supports were calculated based on 1,000 bootstrap replications. A total of 64 sequences of close relatives were downloaded from the NCBI GenBank, and combined with sequences of the 66 endophytic fungi isolated in the present work for phylogenetic tree construction. Two wood decay macrofungi namely *Schizophyllum commune* (phylum Basidiomycota, family Schizophyllaceae) and *Phellinus gabonensis* (phylum Basidiomycota, family Hymenochaetaceae) were included as out-group.

### Antagonism assay

Endophytic fungal isolates were cultivated on PDA plates at 26°C for 7 days. The antagonistic activity was evaluated through the dual culture assay against *C. fimbriata*. The pathogenic *C. fimbriata* (FRIM1162) isolate used in this study was isolated from a infected *Acacia mangium* (Syazwan et al., 2021) and maintained at 27°C on PDA media at the Mycology & Pathology Unit, Forest Research Institute Malaysia (FRIM). Briefly, a fungal disc of 5 mm in diameter was taken from *C. fimbriata*, and placed 3 cm from the margin of the PDA plate (9 cm in diameter). Next, a 5 mm disc of the endophytic fungus was placed 3 cm from the margin of the PDA plate, and directly opposite of the *C. fimbriata* disc. Inoculated PDA plates were incubated at room temperature for 7 days. PDA plates inoculated with *C. fimbriata* in the absence of endophytic fungus served as negative controls. The assay was performed in triplicates. Observations were carried out for 6 days, after which the mycelial radial growth of test pathogen (*C. fimbriata*) on a control plate (rl) and in the presence of the antagonistic fungus (r2) were measured, and the percentage inhibition (I%) in mycelial growth was calculated as: I% = [(r1 – r2) / r1] × 100 (Hajieghrari et al., 2008). The I% data were analysed statistically with ANOVA using the SAS statistical software. To examine the significance between endophytic fungal isolates, Fisher’s LSD was performed at *p* ≤ 0.05.

## Results

### Identification of endophytic fungi

A total of 66 endophytic fungal isolates were successfully isolated from different parts of healthy *A. mangium* (Table 1); 21 from leaf, 12 from petiole, 13 from stem, nine from root, six from flower, and five from fruit. Correspondingly, 66 isolates were successfully amplified using primers ITS1 and ITS4. The endophytic fungal isolates mostly belonged to Ascomycota (95.5%) followed by Zygomycota (4.5%) based on the BLAST searches analysis (Table 2). For Ascomycota, 13 genera were identified; *Trichoderma* (28.6%), *Nigrospora* (28.6%), *Pestalotiopsis* (12.7%), *Lasiodiplodia* (9.5%), *Aspergillus* (6.3%), *Sordariomycetes* (3%), and genera that were represented by a single isolate were *Neopestalotiopsis*, *Pseudopestalotiopsis*, *Eutiarosporella*, *Curvularia*, *Fusarium*, *Penicillium*, and *Hypoxylon*. Only *Blakeslea* sp. (4.5%) of Zygomycota was identified in the present work (Table 1). All the fungal ITS rDNA sequences exhibited high similarity with existing sequences in the NCBI database (Table 1).

**Table 1:** Endophytic fungi isolated from different plant part of healthy *Acacia mangium*.

Plant part	Individual nnumber														Total
Fruit	1			2				1						1	5
Flower		1						2			1		1	1	6
Leaf	4			2		1		4	1	1	8				21
Petiole	1		1	1			1	4			4				12
Stem	2			1	1	1		4			3	1			13
Root						2		3			2	1		1	9
Total	8	1	1	6	1	4	1	18	1	1	18	2	1	3	66
	Pestalotiopsis	Pseudopestalotiopsis	Neopestalotiopsis	Lasiodiplodia	Eutiarosporella	Aspergillus	Penicillium	Trichoderma	Fusarium	Curvularia	Nigrospora	Sordariomycetes	Hypoxylon	Blakeslea	

**Table 2:** Percentage of identity matches of 66 fungal isolates from different plant parts of Acacia mangium based on ITS sequences using BLAST analyses, and their percentage of inhibition against *Ceratocystis fimbriata*.

No.	Endophytic isolate ID	Plant part	Inhibition activities (%) (mean ± standard error)	GenBank Accession number	ITS region				
					Match identity (%)	E-value	Identification in GenBank	BLAST match in GenBank	Phylum, Class, Family
1	AC 1R	Root	55 ± 0.58	MW254902	99.28	0	*Blakeslea trispora*	HQ248186	Zygomycota, Zygomycetes, Choanephoraceae
2	AC 2R	Root	0 ± 0.00	MW254903	99.63	0	*Trichoderma gamsii*	KX009501	Ascomycota, Sordariomycetes, Hypocreaceae
3	AC 3R	Root	0 ± 0.00	MW254904	100	0	*Aspergillus aculeatinus*	MK281555	Ascomycota, Eurotiomycetes, Trichocomaceae
4	AC 4R	Root	44 ± 2.08	MW254905	99.38	0	*Nigrospora sphaerica*	MH368102	Ascomycota, Sordariomycetes, Trichosphaeriales
5	AC 5R	Root	20 ± 0.00	MW254913	99.21	0	*Aspergillus niger*	MN474007	Ascomycota, Eurotiomycetes, Trichocomaceae
6	AC 6R	Root	8.88 ± 0.66	MW254916	99.63	0	*Trichoderma spirale*	MN227543	Ascomycota, Sordariomycetes, Hypocreaceae
7	AC 7R	Root	14.28 ± 0.43	MW254942	99.17	0	*Sordariomycetes* sp.	JQ759985	Ascomycota, Sordariomycetes,
8	AC 8R	Root	25 ± 2.89	MW254956	99.58	0	*Nigrospora oryzae*	MN382281	Ascomycota, Sordariomycetes, Trichosphaeriales
9	AC 9R	Root	58.33 ± 5.02×10^15^	MW254964	99.81	0	*Trichoderma* sp.	MK870905	Ascomycota, Sordariomycetes, Hypocreaceae
10	AC 1S	Stem	58.33 ± 5.02 ×10^15^	MW254907	99.81	0	*Trichoderma koningiopsis*	KY807125	Ascomycota, Sordariomycetes, Hypocreaceae
11	AC 2S	Stem	33.33 ± 0.29	MW254909	99.79	0	*Nigrospora sphaerica*	KJ572188	Ascomycota, Sordariomycetes, Trichosphaeriales
12	AC 3S	Stem	0 ± 0.00	MW254914	99.63	0	*Pestalotiopsis vismiae*	KP747709	Ascomycota, Sordariomycetes, Sporocadaceae
13	AC 4S	Stem	0 ± 0.00	MW254920	99.81	0	*Pestalotiopsis* sp.	KY413701	Ascomycota, Sordariomycetes, Sporocadaceae
14	AC 5S	Stem	45.45 ± 5.02 ×10^15^	MW254924	99.45	0	*Trichoderma* sp.	MK870688	Ascomycota, Sordariomycetes, Hypocreaceae
15	AC 6S	Stem	20 ± 3.61	MW254925	99.15	0	*Lasiodiplodia theobromae*	GQ502461	Ascomycota, Dothideomycetes, Botryosphaeriaceae
16	AC 7S	Stem	45 ± 0.00	MW254931	99.25	0	*Trichoderma gamsii*	KX009501	Ascomycota, Sordariomycetes, Hypocreaceae
17	AC 8S	Stem	40 ± 0.00	MW254937	99.59	0	*Nigrospora oryzae*	MN38228	Ascomycota, Sordariomycetes, Trichosphaeriales
18	AC 9S	Stem	0 ± 0.00	MW254940	99.63	0	*Trichoderma ovalisporum*	FJ442652	Ascomycota, Sordariomycetes, Hypocreaceae
19	AC 10S	Stem	0 ± 0.00	MW254944	99.8	0	*Aspergillus niger*	MN559950	Ascomycota, Eurotiomycetes, Trichocomaceae
20	AC 11S	Stem	45.45 ± 2.60	MW254951	100	0	*Sordariomycetes* sp.	KC178665	Ascomycota, Sordariomycetes,
21	AC 12S	Stem	14.28 ± 0.30	MW254954	97.98	0	*Eutiarosporella* sp.	KX464132	Ascomycota, Dothideomycetes, Botryosphaeriales
22	AC 13S	Stem	0 ± 0.00	MW254959	100	0	*Nigrospora* sp.	MT556677	Ascomycota, Sordariomycetes, Trichosphaeriales
23	AC 1 l	Leaf	55.55 ± 5.02 ×10^15^	MW254906	99.63	0	*Trichoderma gamsii*	KM103313	Ascomycota, Sordariomycetes, Hypocreaceae
24	AC 2 l	Leaf	37.5 ± 1.44	MW254908	99.38	0	*Nigrospora sphaerica*	MN625838	Ascomycota, Sordariomycetes, Trichosphaeriales
25	AC 3 l	Leaf	45.45 ± 0.75	MW254910	99.81	0	*Trichoderma gamsii*	KX009501	Ascomycota, Sordariomycetes, Hypocreaceae
26	AC 4 l	Leaf	16.67 ± 9.53	MW254918	100	0	*Curvularia pandanicola*	MH275056	Ascomycota, Dothideomycetes, Pleosporaceae
27	AC 5 l	Leaf	16.67 ± 0.00	MW254919	99.63	0	*Pestalotiopsis microspora*	MT597837	Ascomycota, Sordariomycetes, Sporocadaceae
28	AC 6 l	Leaf	45.45 ± 1.16	MW254921	99.81	0	*Pestalotiopsis microspora*	EU137910	Ascomycota, Sordariomycetes, Sporocadaceae
29	AC 7 l	Leaf	58.3 ± 5.02 ×10^15^	MW254922	98.77	0	*Nigrospora oryzae*	MN382281	Ascomycota, Sordariomycetes, Trichosphaeriales
30	AC 8 l	Leaf	28.57 ± 2.51 ×10^15^	MW254923	99.58	0	*Fusarium chlamydosporum*	MT448890	Ascomycota, Sordariomycetes, Nectriaceae
31	AC 9 l	Leaf	0 ± 0.00	MW254926	99.38	0	*Nigrospora sphaerica*	MN566004	Ascomycota, Sordariomycetes, Trichosphaeriales
32	AC 10 l	Leaf	30 ± 5.77	MW254934	99.57	0	*Lasiodiplodia theobromae*	KF293981	Ascomycota, Dothideomycetes, Botryosphaeriaceae
33	AC 11 l	Leaf	12.5 ± 6.93	MW254936	99.63	0	*Trichoderma koningiopsis*	JQ617301	Ascomycota, Sordariomycetes, Hypocreaceae
34	AC 12 l	Leaf	0 ± 0.00	MW254938	99.44	0	*Pestalotiopsis neglecta*	MN006391	Ascomycota, Sordariomycetes, Sporocadaceae
35	AC 13 l	Leaf	22.22 ± 0.00	MW254939	99.62	0	*Trichoderma gamsii*	KX009501	Ascomycota, Sordariomycetes, Hypocreaceae
36	AC 14 l	Leaf	0 ± 0.00	MW254943	99.58	0	*Nigrospora oryzae*	JX966549	Ascomycota, Sordariomycetes, Trichosphaeriales
37	AC 15 l	Leaf	33.33 ± 1.59	MW254945	99.79	0	*Nigrospora* sp.	MT561433	Ascomycota, Sordariomycetes, Trichosphaeriales
38	AC 16 l	Leaf	45.45 ± 5.02 ×10^15^	MW254946	99.58	0	*Lasiodiplodia theobromae*	MK696043	Ascomycota, Dothideomycetes, Botryosphaeriaceae
39	AC 17 l	Leaf	40 ± 5.77	MW254948	99.43	0	*Pestalotiopsis vismiae*	KP747709	Ascomycota, Sordariomycetes, Sporocadaceae
40	AC 18 l	Leaf	25 ± 0.00	MW254949	99.59	0	*Nigrospora sphaerica*	MT043797	Ascomycota, Sordariomycetes, Trichosphaeriales
41	AC 19 l	Leaf	0 ± 0.00	MW254950	99.8	0	*Aspergillus aculeatus*	KJ605160	Ascomycota, Eurotiomycetes, Trichocomaceae
42	AC 20 l	Leaf	40 ± 5.77	MW254962	99.59	0	*Nigrospora sphaerica*	MH368102	Ascomycota, Sordariomycetes, Trichosphaeriales
43	AC 21 l	Leaf	0 ± 0.00	MW254963	99.58	0	*Nigrospora sphaerica*	MT561433	Ascomycota, Sordariomycetes, Trichosphaeriales
44	AC 1P	Petiole	0 ± 0.00	MW254917	99.81	0	*Trichoderma crissum*	MK911703	Ascomycota, Sordariomycetes, Hypocreaceae
45	AC 2P	Petiole	0 ± 0.00	MW254932	99.79	0	*Nigrospora sphaerica*	MT561433	Ascomycota, Sordariomycetes, Trichosphaeriales
46	AC 3P	Petiole	50 ± 4.91	MW254933	97.68	0	*Nigrospora sphaerica*	MN795570	Ascomycota, Sordariomycetes, Trichosphaeriales
47	AC 4P	Petiole	58.33 ± 5.02 ×10^15^	MW254935	99.38	0	*Nigrospora sphaerica*	KJ572188	Ascomycota, Sordariomycetes, Trichosphaeriales
48	AC 5P	Petiole	45.45 ± 5.02 ×10^15^	MW254947	99.79	0	*Nigrospora sphaerica*	MT561433	Ascomycota, Sordariomycetes, Trichosphaeriales
49	AC 6P	Petiole	45 ± 5.77	MW254952	99.8	0	*Penicillium rolfsii*	MK120600	Ascomycota, Eurotiomycetes, Trichocomaceae
50	AC 7P	Petiole	20 ± 5.77	MW254957	100	0	*Trichoderma longibrachiatum*	FJ462745	Ascomycota, Sordariomycetes, Hypocreaceae
51	AC 8P	Petiole	30 ± 5.77	MW254958	99.37	0	*Neopestalotiopsis cubana*	LC521857	Ascomycota, Sordariomycetes, Pestalotiopsidaceae
52	AC 9P	Petiole	45.45 ± 5.02 ×10^15^	MW254961	99.79	0	*Pestalotiopsis* sp.	JN116590	Ascomycota, Sordariomycetes, Sporocadaceae
53	AC 10P	Petiole	45.45 ± 5.02 ×10^15^	MW254965	99.57	0	*Lasiodiplodia theobromae*	MT075441	Ascomycota, Dothideomycetes, Botryosphaeriaceae
54	AC 11P	Petiole	14.28 ± 0	MW254966	70.1	4.00E-20	*Trichoderma* sp.	GU973813	Ascomycota, Sordariomycetes, Hypocreaceae
55	AC 12P	Petiole	20 ± 5.77	MW254967	99.44	0	*Trichoderma koningiopsis*	MT102395	Ascomycota, Sordariomycetes, Hypocreaceae
56	AC 1F	Flower	8.88 ± 0.00	MW254911	99.28	0	*Blakeslea trispora*	HQ248186	Zygomycota, Zygomycetes, Choanephoraceae
57	AC 2F	Flower	8.88 ± 5.14	MW254915	94.87	0	*Hypoxylon monticulosum*	KY610404	Ascomycota, Sordariomycetes, Hypoxylaceae
58	AC 3F	Flower	58.33 ± 5.02 ×10^15^	MW254927	100	0	*Nigrospora sphaerica*	MT561433	Ascomycota, Sordariomycetes, Trichosphaeriales
59	AC 4F	Flower	40 ± 0.00	MW254941	99.62	0	*Trichoderma longibrachiatum*	MH745146	Ascomycota, Sordariomycetes, Hypocreaceae
60	AC 5F	Flower	45.45 ± 2.60	MW254953	99.59	0	*Pseudopestalotiopsis theae*	KX401429	Ascomycota, Sordariomycetes, Pestalotiopsidaceae
61	AC 6F	Flower	45.45 ± 5.02 ×10^15^	MW254955	100	0	*Trichoderma koningiopsis*	JQ278013	Ascomycota, Sordariomycetes, Hypocreaceae
62	AC 1 U	Fruit	8.88 ± 0.00	MW254912	100	0	*Pestalotiopsis microspore*	EU137910	Ascomycota, Sordariomycetes, Sporocadaceae
63	AC 2 U	Fruit	69.23 ± 0.00	MW254928	99.79	0	*Lasiodiplodia theobromae*	MK696044	Ascomycota, Dothideomycetes, Botryosphaeriaceae
64	AC 3 U	Fruit	25 ± 2.89	MW254929	98.39	0	*Blakeslea trispora*	HQ248186	Zygomycota, Zygomycetes, Choanephoraceae
65	AC 4 U	Fruit	45 ± 2.89	MW254930	99.79	0	*Lasiodiplodia venezuelensis*	MH865369	Ascomycota, Dothideomycetes, Botryosphaeriaceae
66	AC 5 U	Fruit	16.16 ± 0.00	MW254960	100	0	*Trichoderma harzianum*	MF537642	Ascomycota, Sordariomycetes, Hypocreaceae

**Figure 1:**
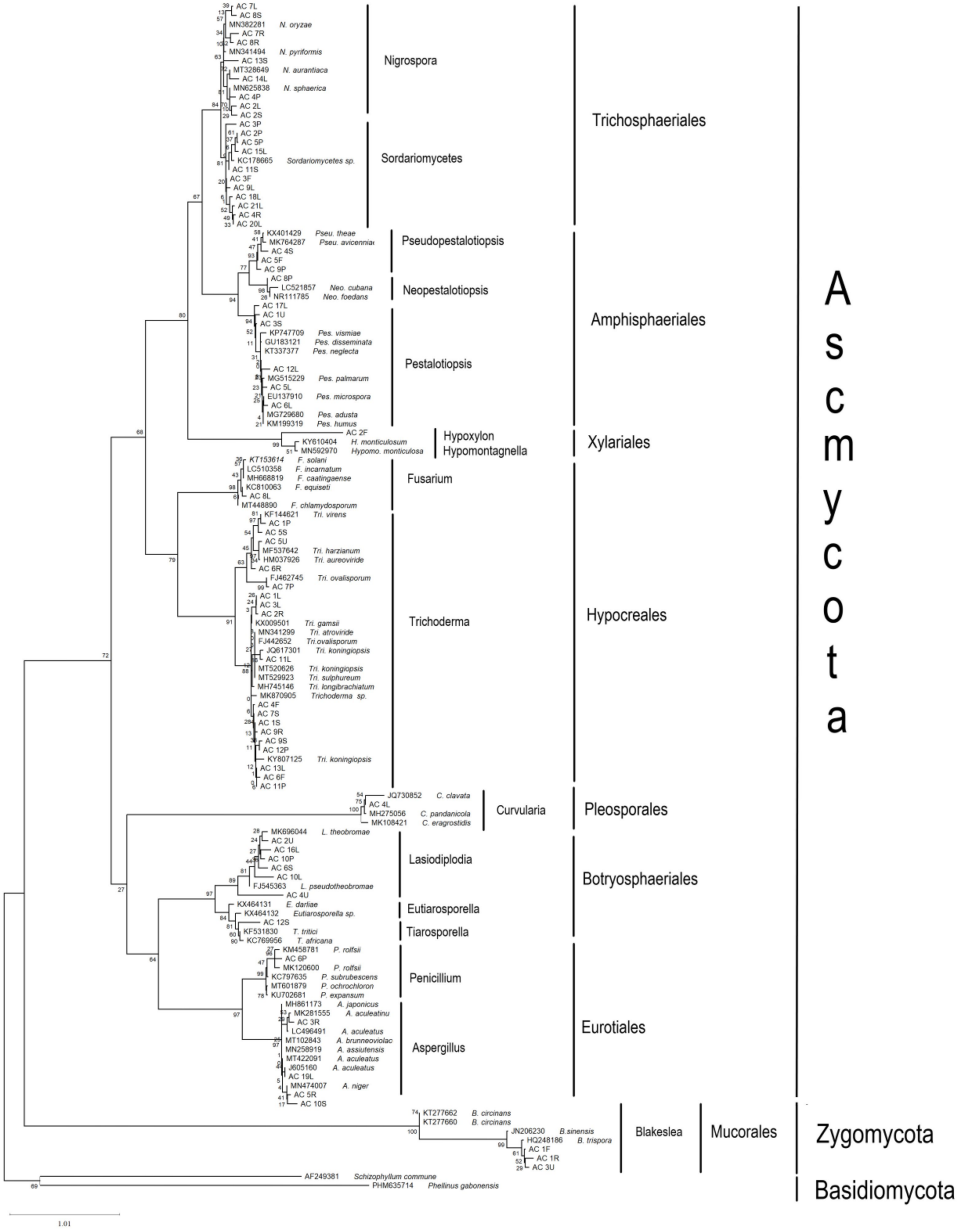
Maximum likelihood (ML) phylogenetic tree based on rDNA ITS sequences of endophytic fungal isolates and fungal ITS sequences from the GenBank. ML tree was constructed using the Kimura 2-parameter (K2) model and gamma distributed (+G) model. All positions containing gaps and missing data were included for analysis. Clade supports were calculated based on 1,000 bootstrap.

The ITS sequences obtained in the present work were deposited in the NCBI GenBank (MW254902 - MW254967) for future reference. A total of 66 sequences of close relatives were downloaded from the NCBI GenBank, and combined with sequences of the 66 endophytic fungi for phylogenetic tree construction (Figure 1). Nine different orders were observed, of which six belonged to Ascomycota (Amphisphaeriales, Brotryosphaerialase, Eurotiales, Hypocreales, Pleosporales and Trichosphaeriales), one belonged to Zygomycota, and two belonged to Basidiomycota (out-group). Most of the endophytic fungal isolates clustered under the order Trichosphaeriales (20 isolates) belonged to genus *Nigrospora*, and under the order Hypocreales (19 isolates) belonged to genera *Fusarium* and *Trichoderma*. Tables 3 and 4 summarises these results.

**Table 3:** Endophytic fungal orders from the phylum Ascomycota.

No.	ID	GenBank Accession no.	Plant part	Amphisphaeriales	
1	AC 3S	MW254914	Stem	*Pestalotiopsis vismiae*	*Pestalotiopsis* clade (94% bootstrap)
2	AC 4S	MW254920	Stem	*Pestalotiopsis* sp.	
3	AC 9P	MW254961	Petiole	*Pestalotiopsis* sp.	
4	AC 5L	MW254919	Leaf	*Pestalotiopsis microspora*	
5	AC 6L	MW254921	Leaf	*Pestalotiopsis microspora*	
6	AC 12L	MW254938	Leaf	*Pestalotiopsis neglecta*	
7	AC 1U	MW254912	Fruit	*Pestalotiopsis microspora*	
8	AC 5F	MW254953	Flower	*Pseudopestalotiopsis theae*	
9	AC 17L	MW254948	Leaf	*Pestalotiopsis vismiae*	*Pseudopestalotiopsis* clade (77% bootstrap)
10	AC 8P	MW254958	Petiole	*Neopestalotiopsis cubana* Brotryosphaerialase	*Neopestalotiopsis* clade (77% bootstrap)
11	AC 6S	MW254925	Stem	*Lasiodiplodia theobromae*	*Lasiodiplodia* clade (97% bootstrap)
12	AC 10P	MW254965	Petiole	*Lasiodiplodia theobromae*	
13	AC 10L	MW254934	Leaf	*Lasiodiplodia theobromae*	
14	AC 16L	MW254946	Leaf	*Lasiodiplodia theobromae*	
15	AC 2U	MW254928	Fruit	*Lasiodiplodia theobromae*	
16	AC 4U	MW254930	Fruit	*Lasiodiplodia venezuelensis*	
17	AC 12S	MW254954	Stem	*Eutiarosporella* sp. Eurotiales	*Eutiarosporella* clade (97% bootstrap)
18	AC 10S	MW254944	Stem	*Aspergillus niger*	*Aspergillus* clade (97% bootstrap)
19	AC 3R	MW254904	Root	*Aspergillus aculeatinus*	
20	AC 5R	MW254913	Root	*Aspergillus niger*	
21	AC 19L	MW254950	Leaf	*Aspergillus aculeatus*	
22	AC 6P	MW254952	Petiole	*Penicillium rolfsii* Hypocreales	*Penicillium* clade (97% bootstrap)
23	AC 2R	MW254903	Root	*Trichoderma gamsii*	*Trichoderma* clade (95% bootstrap)
24	AC 6R	MW254916	Root	*Trichoderma spirale*	
25	AC 9R	MW254964	Root	*Trichoderma* sp.	
26	AC 1S	MW254907	Stem	*Trichoderma koningiopsis*	
27	AC 5S	MW254924	Stem	*Trichoderma* sp.	
28	AC 7S	MW254931	Stem	*Trichoderma gamsii*	
29	AC 9S	MW254940	Stem	*Trichoderma ovalisporum*	
30	AC 1P	MW254917	Petiole	*Trichoderma crissum*	
31	AC 7P	MW254957	Petiole	*Trichoderma longibrachiatum*	
32	AC 11P	MW254966	Petiole	*Trichoderma* sp.	
33	AC 12P	MW254967	Petiole	*Trichoderma koningiopsis*	
34	AC 1L	MW254906	Leaf	*Trichoderma gamsii*	
35	AC 3L	MW254910	Leaf	*Trichoderma gamsii*	
36	AC 11L	MW254936	Leaf	*Trichoderma koningiopsis*	
37	AC 13L	MW254939	Leaf	*Trichoderma gamsii*	
38	AC 4F	MW254941	Flower	*Trichoderma longibrachiatum*	
39	AC 5U	MW254960	Fruit	*Trichoderma harzianum*	
40	AC 6F	MW254955	Fruit	*Trichoderma koningiopsis*	
41	AC 8L	MW254923	Leaf	*Fusarium chlamydosporum* Pleosporales	*Fusarium* clade (95% bootstrap)
42	AC 4L	MW254918	Leaf	*Curvularia pandanicola*	*Curvularia* clade (95% bootstrap)
				Hypocreales	
43	AC 4R	MW254905	Root	*Nigrospora sphaerica*	*Nigrospora* and *Sordariomycete polytomy* clade (95% bootstrap)
44	AC 8R	MW254956	Root	*Nigrospora oryzae*	
45	AC 2S	MW254909	Stem	*Nigrospora sphaerica*	
46	AC 8S	MW254937	Stem	*Nigrospora oryzae*	
47	AC 13S	MW254959	Stem	*Nigrospora* sp.	
48	AC 2P	MW254932	Petiole	*Nigrospora sphaerica*	
49	AC 3P	MW254933	Petiole	*Nigrospora sphaerica*	
50	AC 4P	MW254935	Petiole	*Nigrospora sphaerica*	
51	AC 5P	MW254947	Petiole	*Nigrospora sphaerica*	
52	AC 2L	MW254908	Leaf	*Nigrospora sphaerica*	
53	AC 7L	MW254922	Leaf	*Nigrospora oryzae*	
54	AC 9L	MW254926	Leaf	*Nigrospora sphaerica*	
55	AC 14L	MW254943	Leaf	*Nigrospora oryzae*	
56	AC 15L	MW254945	Leaf	*Nigrospora* sp.	
57	AC 18L	MW254949	Leaf	*Nigrospora sphaerica*	
58	AC 20L	MW254962	Leaf	*Nigrospora sphaerica*	
59	AC 21L	MW254963	Leaf	*Nigrospora sphaerica*	
60	AC 3F	MW254927	Flower	*Nigrospora sphaerica*	
61	AC 7R	MW254942	Root	*Sordariomycetes* sp.	
62	AC 11S	MW254951	Stem	*Sordariomycetes* sp. Hypocreales	
63	AC 2F	MW254915	Flower	*Hypoxylon monticulosum*	*Hypoxylon* clade (99% bootstrap)

**Table 4:** Endophytic fungal order from the phylum Zygomycota.

No.	ID	GenBank Accession number	Plant part	Mucorales	
1	AC 1R	MW254902	Root	*Blakeslea trispora*	*Blakeslea* clade (99% bootstrap)
2	AC 1F	MW254911	Flower	*Blakeslea trispora*	
3	AC 3U	MW254929	Fruit	*Blakeslea trispora*	

### Antagonism assay

**Figure 2:**
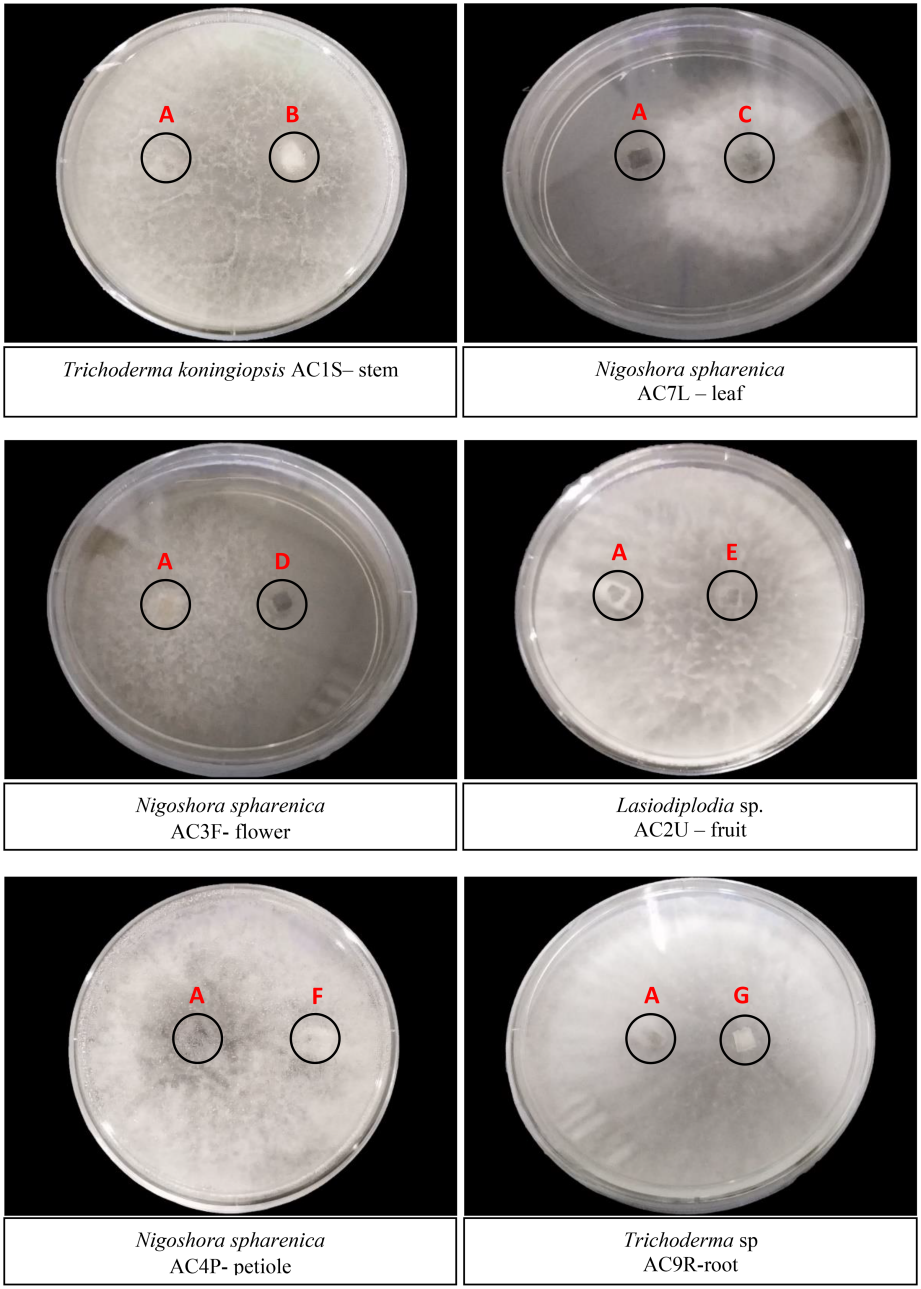
Dual culture plate assay between six endophytic fungal isolates against the pathogen *C. fimbriata* (A). (B) *Trichoderma koningiopsis* AC1S – stem; (C) *Nigoshora spharenica* AC7L – leaf; (D) *Nigoshora spharenica* AC3F - flower; (E) *Lasiodiplodia* sp. AC2U – fruit; (F) *Nigoshora spharenica* AC4P-petiole; (G) *Trichoderma* sp. AC9R-root. The plates were cultivated for 5 days at 27°C. Radial growths were measured and interaction were observed.

All 66 endophytic fungal isolates were tested in the antagonism assay against *C. fimbriata.* After 5 days of incubation, six fungal isolates namely *Trichoderma koningiopsis* (AC 1S) stem, *Nigrospora oryzae* (AC 7L) leaf, *Nigrospora sphaerica* (AC 3F) flower, *Lasiodiplodia* sp. (AC 2 U) fruit, *Nigrospora sphaerica* (AC 4P) petiole, and *Trichoderma* sp. (AC 9R) root were observed to exhibit stronger inhibition where the mycelia of the antagonists had breached into *C. fimbriata* colony (Figure 2). Of these, four fungal isolates namely *T. koningiopsis* (AC 1S) stem, *Lasiodiplodia* sp. (AC 2 U) fruit, *N. sphaerica* (AC 4P) petiole, and *Trichoderma* sp. (AC 9R) root colonised almost 99% of the culture plate. Although *N. sphaerica* (AC 7 l) leaf and *N. sphaerica* (AC 3F) flower did not colonise the entire culture plate, there was no growth of *C. fimbriata* observed.

**Figure 3:**
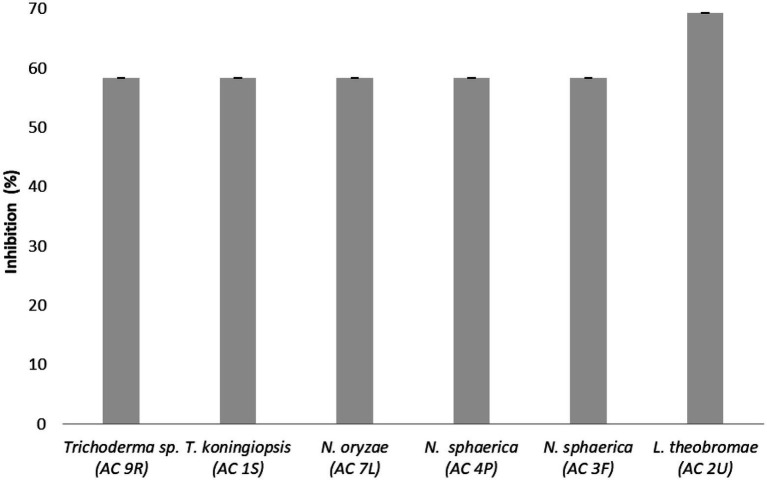
Inhibition percentages (I%) of endophytic fungi against the pathogen *Ceratocystis fimbriata* in dual culture assay. Data are mean ± standard error (SE) of triplicates.

The inhibition percentages (I%) of endophytic fungi against the pathogen *C. fimbriata* in dual culture assay are shown in Figure 3. *Lasiodiplodia* sp. (AC 2 U) isolated from fruit recorded the highest I% (69.23%), followed by *Trichoderma* sp. (AC 9R) isolated from root, *Nigrospora sphaerica (*AC 4P) isolated from petiole, *Nigrospora sphaerica* (AC 3F) isolated from flower, *Trichoderma koningiopsis* (AC 1S) isolated from stem, and *Nigrospora oryzae* (AC 7L) isolated from leaf with value 58.33%, respectively.

**Figure 4:**
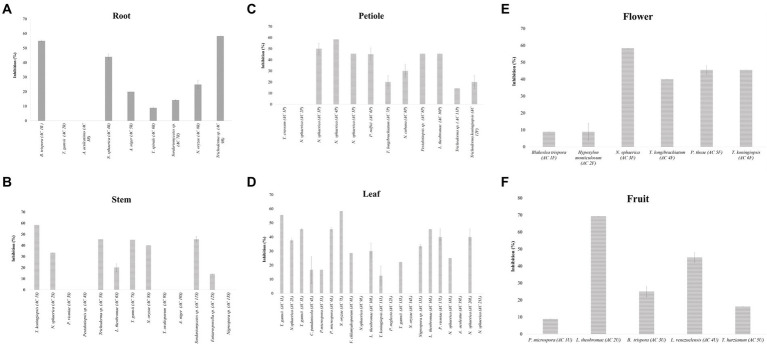
The inhibition percentages (I%) of endophytic fungi isolated from root **(A)**, stem **(B)**, petiole **(C)**, leaf **(D)**, flower **(E)**, and fruit **(F)** against the pathogen *Ceratocystis fimbriata*. Data are mean ± standard error (SE) of triplicates. Means followed by the same letter in each group are not significantly different at α = 0.05 according to DuncanLSD.

Thirteen endophytic fungi from various plant parts of *A. mangium* showed no inhibition against *C. fimbriata* (Figure 4) namely *A. aculeatinus* (AC 3R) isolated from root, *A. aculeatus* (AC 19L) isolated from leaf, *A. niger* (AC 10S) isolated from stem, *N. oryzae* (AC 14L) isolated from leaf, *Nigrospora* sp. (AC 13S) isolated from stem, *N. sphaerica* (AC 9L) isolated from leaf, *N. sphaerica* (AC 2P) isolated from petiole, *P. neglecta* (AC 12L) isolated from leaf, *Pestalotiopsis* sp. (AC 4S) isolated from stem, *P. vismiae* (AC 3S) isolated from stem, *T. crissum* (AC 1P) isolated from petiole, *T. gamsii* (AC 2R) isolated from root, and *T. ovalisporum* (AC 9S) isolated from stem.

### Diversity of endophytic fungi

Endophytic fungi are ubiquitous, and every plant species examined to date have been found colonised by them (Arnold et al., 2001). A single plant species may harbour hundreds of endophytes which may inhabit all available tissues, including leaves, petioles, stems, twigs, barks, xylems, roots, fruits, flowers, and seeds (Chapela and Boddy, 1988; Fisher et al., 1993; Saikkonen et al., 1998; Jena and Tayung, 2013). In the present work, endophytic fungi were isolated from different plant parts of *A. mangium* with the highest number of isolates found in leaf and dominated by the genera *Trichoderma* and *Nigrospora*. *Trichoderma* spp. were present in all plant parts, while *Nigrospora* spp. were present in all but fruit. In total, 66 endophytic fungal isolates were obtained from different plant parts of *A. mangium*.

*Trichoderma* and *Nigrospora* have also been reported as endophytes in other plants such as *Rauvolfia serpentine*, *Prosopis cineraria*, and *Piper nigrum* (Gehlot et al., 2008; Dutta et al., 2014; Sopialena et al., 2018). *Trichoderma* is also found in many ecosystems, and can reduce the severity of plant diseases by inhibiting the plant pathogens in the soil through their highly potent antagonistic and mycoparasitic activities (Hermosa et al., 2012). Moreover, as revealed by research in recent decades, some *Trichoderma* strains can interact directly with roots, thus increasing plant growth potential, resistance to disease, and tolerance to abiotic stresses (Mastouri et al., 2010; Hermosa et al., 2012; Brotman et al., 2013). *Nigrospora* is also a beneficial member of the foliar endophytic community due to its mutualistic existence with their host plants, and having a potential for biological control strategies (Zakaria et al., 2016). Other than *Nigrospora*, *Pestalotiopsis* also is a beneficial member of the foliar endophytic community due to its ability to switch its nutritional mode, thus able to stay as an endophyte or switch to saprophyte when necessary (Douanla-Meli et al., 2013; Hamzah et al., 2018). Besides *Trichoderma*, *Nigrospora*, and *Pestalotiopsis*, other fungal genera such as *Lasiodiplodia*, *Sordariomycetes*, and *Aspergillus* have also been reported as predominant endophytic fungi in other plants species (Li et al., 2012; del Castillo et al., 2016), and have an antagonism ability (Chen et al., 2010). *Fusarium* too is a common endophytic fungal genus found in trees (Zakaria et al., 2010). Although it is widely available in most tropical plants investigated in past studies (Warman and Aitken, 2018), we recorded a low isolation frequency of *Fusarium*. Our finding also revealed lesser-known fungal genera, namely *Eutiarosporella*, *Curvularia*, *Glomerella*, and *Hypoxylon* in *A. mangium*.

In the present work, ITS sequences identified 63 endophytic fungal isolates from the phylum Ascomycota, and three from Zygomycota. The phylum Ascomycota has been reported to be the most common endophytic fungal phylum when isolated using standard isolation protocols (Koukol et al., 2012; Hamzah et al., 2018). Fungi from the phylum Zygomycota have been reported to be culture-method dependent (Crozier et al., 2006; Hamzah et al., 2018), which might explain the small isolate number reported in the present work. Comparative studies also show that only a small fraction of microorganisms in nature can be cultured using conventional microbiological techniques (Amann et al., 1995). There are many factors that can affect the microbial viability under laboratory conditions, for example the lack of knowledge about their nutritional requirements.

### Antagonism activities against *Ceratocystis fimbriata*

Fungal antagonism can manifest in many ways such as nutrition competition, niche exclusion, mycoparasitism, and the production of extracellular metabolites (Siameto et al., 2010). These metabolites, especially antibiotics and lytic enzymes, have been widely applied in various fields like crop-pathogen controls. Endophytic microorganisms isolated from plants can produce various novel bioactive metabolites (Ramasamy et al., 2010). The bioactive metabolites produced by plants, microorganisms, and organisms are useful for the discovery and development of new drugs.

In the present work, *Lasiodiplodia* sp., *T. koningiopsis*, *N. sphaerica* and *Trichoderma* sp. successfully inhibited the pathogen *C. fimbriata* in the dual culture assay. The ability to out-grow the pathogen *in vitro* suggested that these fungi competed for the space and nutrient with the pathogen. In theory, biological agents with antifungal properties are known to secrete certain enzymes which break down their competitors’ cell wall, thus restricting their growth (Sharon et al., 2001). The antagonism displayed by *Lasiodiplodia* sp. was more aggressive as compared to other endophytic fungi (Figure 3). This could be attributed to the production of lytic enzymes by *Lasiodiplodia* sp. (Anitha and Rabeeth, 2010). The antagonism displayed by *Lasiodiplodia* sp., *T. koningiopsis*, *N. sphaerica* and *Trichoderma* sp. could also be explained by their secretion of secondary metabolites into the growth medium, as well as nutrient depletion in the growth medium (Robinson et al., 2014). The antagonism displayed might also be influenced by the antibiotics or hydrolytic enzymes they produced (Kamala and Indira, 2011). The difference in antagonism magnitude observed in the present work could also be dependent on specific fungal species (Kai et al., 2007). Previously, *Lasiodiplodia* sp. from the flower of *Viscum coloratum* also exhibited antimicrobial activity which could be due to the presence of cyclo-(Trp-Ala), ICA, indole-3-carbaldehyde, mullein, and 2-phenylethano in their extract (Qian et al., 2014). *Lasiodiplodia* sp. isolated from the twig of *Aegle marmelos* has also been shown to have *in vitro* fibrinolytic activities (Meshram and Saxena, 2016). Another plant parts such as bark and leaf of *Terminalia* sp. has also been isolated with *Lasiodiplodia* sp. which not only exhibited antimicrobial and antioxidant activities, but also aided the plant to withstand stressful environmental conditions (Patil et al., 2014).

## Conclusion

Diversity of endophytic fungi were successfully isolated from different parts of *A. mangium*, with *Trichoderma* spp. being the most prevalent, and were isolated from all six plant parts. Against *C. fimbriata*, the crude extracts from *Trichoderma* spp.*, N. sphaerica*, and *Lasiodiplodia* sp. exhibited strong inhibition in the dual culture assay. Thus, it can be concluded that certain endophytic fungi of *A. mangium* have the potential to be harnessed as anti-Ceratocystis agent in future biotechnological applications.

## Data availability statement

The datasets presented in this study can be found in online repositories. The names of the repository/repositories and accession number(s) can be found in the article/supplementary material.

## Author contributions

RT designed the study, collected, identified plant materials, and edited the manuscript. RT and RZ conducted the experiments, drafted, and revised the manuscript. Data analysis performed by RT, MA, MM, NS, WAW-M-A, and AH. MH assisted in DNA extraction. RT, MA, MM, NS, and AH supervised. RT acquired funding. All authors contributed to the article and approved the submitted version.

## Funding

The present work was financially supported by Universiti Putra Malaysia under the Putra Grant Scheme (GP-IPM/2017/9565600).

## Conflict of interest

The authors declare that the research was conducted in the absence of any commercial or financial relationships that could be construed as a potential conflict of interest.

## Publisher’s note

All claims expressed in this article are solely those of the authors and do not necessarily represent those of their affiliated organizations, or those of the publisher, the editors and the reviewers. Any product that may be evaluated in this article, or claim that may be made by its manufacturer, is not guaranteed or endorsed by the publisher.

## Footnotes

^1^ https://blast.ncbi.nlm.nih.gov/Blast.cgi
